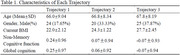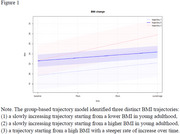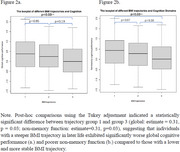# Body‐shape fluctuation through four decades of lifespan and neurocognitive functions in older adults without cognitive impairment

**DOI:** 10.1002/alz70861_108504

**Published:** 2025-12-23

**Authors:** Yifan Yan, Xuhao Zhao, Ting Pang, Yaping Zhang, Renwei Chen, Changzheng Yuan, Xin Xu

**Affiliations:** ^1^ School of Public Health, the Second Affiliated Hospital of School of Medicine, Zhejiang University, Hangzhou, Zhejiang China; ^2^ The Key Laboratory of Intelligent Preventive Medicine of Zhejiang Province, Hangzhou, Zhejiang China; ^3^ Key Laboratory of Intelligent Preventive Medicine of Zhejiang Province, Hangzhou, Zhejiang China; ^4^ Zhejiang University School of Medicine, Hangzhou, Zhejiang China; ^5^ Harvard T. H. Chan School of Public Health, Boston, MA USA; ^6^ Memory, Ageing, and Cognition Centre (MACC), Department of Pharmacology, Yong Loo Lin School of Medicine, National University of Singapore, Singapore Singapore

## Abstract

**Background:**

Dementia poses a growing global health burden, with early prevention during the preclinical stage being key. Body Mass Index (BMI), a common measure of body composition, has been associated with an increased risk of dementia. However, BMI changes dynamically across the lifespan, and these longitudinal patterns related to the cognitive outcomes remain underexplored. In cognitively intact older adults, understanding how long‐term BMI trajectories relate to cognitive performance is crucial for early intervention strategies. This study investigates the association between BMI trajectories from early to late adulthood and cognitive function using a group‐based trajectory model.

**Method:**

We recruited community‐dwelling Chinese adults aged over 50 years. Participants underwent medical and cognitive assessments. Global cognition and domain‐specific performance were standardized into Z‐scores; those with scores below ‐1.5 SD were excluded. BMI was calculated using measured height and weight at the current time and self‐reported values at ages 25, 40, and 60. Participants with less than three BMI time points were excluded. BMI trajectories were derived using group‐based modeling. Model fit was evaluated with BIC and OCC. Cognitive differences between trajectory groups were analyzed with estimated marginal means, adjusted for age and gender.

**Result:**

Among 265 participants (mean age 67.1±8.4 years; 67.2% female), BMI ranged from 16.5 to 34.2. Among whom 35.84% were overweight (range from 24 to 27.9), whilst 13.96% were obese. A three‐group trajectory model best fit the data (Table 1, Figure 1): Group 1: low starting BMI, slow increase (estimated marginal means=0.29, 95% CI= (0.13, 0.45)); Group 2: higher starting BMI, slow increase (0.22, 95% CI= (0.02, 0.43)); Group 3: high starting BMI, rapid increase (‐0.02, 95% CI= (‐0.20, 0.17)). Group 1 had significantly better global and non‐memory cognition than Group 3 (p = 0.03), with no significant differences between other groups (Figure 2).

**Conclusion:**

The present study spans nearly four decades of longitudinal BMI data in a community‐based cohort, aiming to elucidate the potential impact of lifespan body‐shape fluctuations on late‐life cognitive performance and its specific domains. A steeper BMI increase is associated with worse cognitive performance, suggesting BMI change patterns may offer early warning for cognitive decline.